# Population and Conservation Status of *Buxbaumia viridis* (DC.) Moug. & Nestl. in Romania

**DOI:** 10.3390/plants12030473

**Published:** 2023-01-19

**Authors:** Sorin Ștefănuț, Constanța Mihaela Ion, Tiberiu Sahlean, Gabriela Tamas, Georgiana-Roxana Nicoară, Mihnea Vladimirescu, Ana-Maria Moroșanu, Florența-Elena Helepciuc, Miruna-Maria Ștefănuț, Constantin-Ciprian Bîrsan

**Affiliations:** 1 Institute of Biology Bucharest, Romanian Academy, 296 Splaiul Independenței, P.O. Box 56–53, 060031 Bucharest, Romania; 2 Faculty of Horticulture, University of Agronomic Science & Veterinary Medicine–Bucharest, Bulevardul Mărăşti Nr. 59, 011464 Bucharest, Romania

**Keywords:** bryophytes, threatened species, protected species, nature conservation

## Abstract

The field survey made in the last 20 years revealed that large areas of Romania’s territory are still unexplored concerning moss distribution. The long-term research in natural and mature spruce forest habitats of this country shows that many sites are well protected, a status that is also confirmed by reports of *Buxbaumia viridis*. However, many other sites where this species was identified still lack legal protection. We also generated a potential distribution of the species based on an ensemble model, useful in guiding extensive field surveys and also management and conservation measures. In a country where the volume of wood cut by illegal logging is larger than the legal one, according to official data, it is very important that all habitats for *B. viridis* be included in protected areas. Our paper brings important data to aid in this goal.

## 1. Introduction

*Buxbaumia viridis* (DC.) Moug. & Nestl. is a European protected species, listed in Annex II of the Habitats Directive (Directive 92/43/CEE) [1]. This species is characteristic of natural, mature spruce forests. It grows especially on dead wood, but occasionally it can also be found on soil, provided wood remains exist [2]. It prefers wood in an advanced stage of decay, being found on large fallen spruce trunks, especially near streams, due to its need for high humidity. As a result of its ecology, *B. viridis* is considered an indicator species for spruce forests in good and very good conservation conditions [3] but can also be found in managed forests [2,4].

*B. viridis* is a Least Concern (LC) moss of Europe but is a threatened species in many countries: it is Critically Endangered (CR) in Serbia, Montenegro, and Italy, Endangered (EN) in Finland, Greece, Hungary, Romania and Vulnerable (VU) in Andorra, Great Britain, Spain, Czech Republic, Slovakia and Estonia [5].

According to the Ministry of Environment, Waters and Forests, it is estimated that about 38.6 million m^2^ of wood are logged every year in Romania, but only 18.5 million are legally extracted [6]. Even if Romania has a large area of natural forest, illegal cutting represents the biggest threat to mosses such as *B. viridis.*

*B. viridis* has been known in Romania since the 19th century, and there are many confirmations of the species’ presence on Romanian territory. However, recent research has shown that there are still many areas that have not yet been fully explored, and *B. viridis* can be found there, especially if suitable habitats exist but have not been previously detected.

The first map of the species’ distribution was published by Tarnavschi [7], and country-wide distributions were published by C. Papp [8], E. Plămadă and C. Dumitru [9], G. Mohan [10] and S. Mihăilescu et al. [11]. *B. viridis* was included in the “Endangered” threat category–EN A3c; C1 [12] and has been proposed as a candidate for the European Bryophytes Red List [13].

The distribution range for *B. viridis* in Romania is underestimated, and in our recent field trips, we realized that there is a large area lacking proper surveys. There is also a lack of information on the population size of this species in Romania.

The aims of the current study were to update the distribution range and explore the potential distribution of the species in Romania using species distribution modeling, to establish a baseline population size, and to reevaluate the conservation status in a national area. Moreover, the ensemble models generated can be used to direct future field surveys for this moss species.

## 2. Results

### 2.1. Current Species Distribution

**Figure 1:**
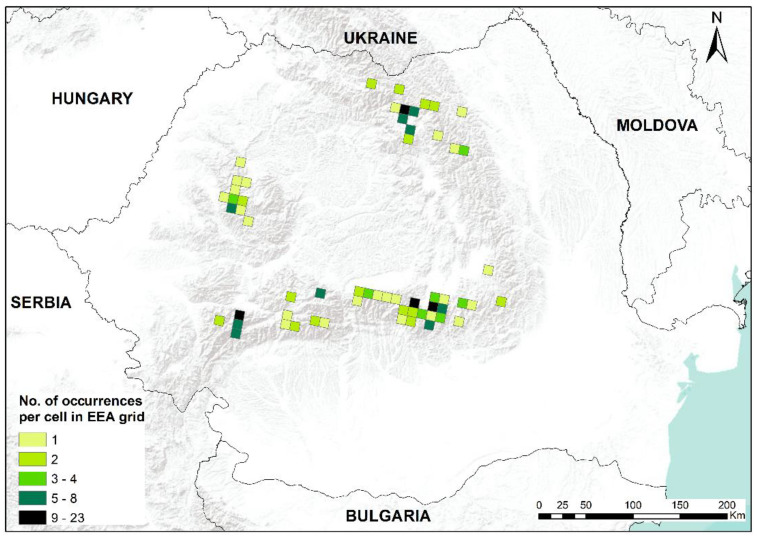
Occurrence of *Buxbaumia viridis* in Romania, EEA10 × 10 km grid resolution. Darker green shades indicate an increased number of occurrences (localities) per cell.

**Figure 2:**
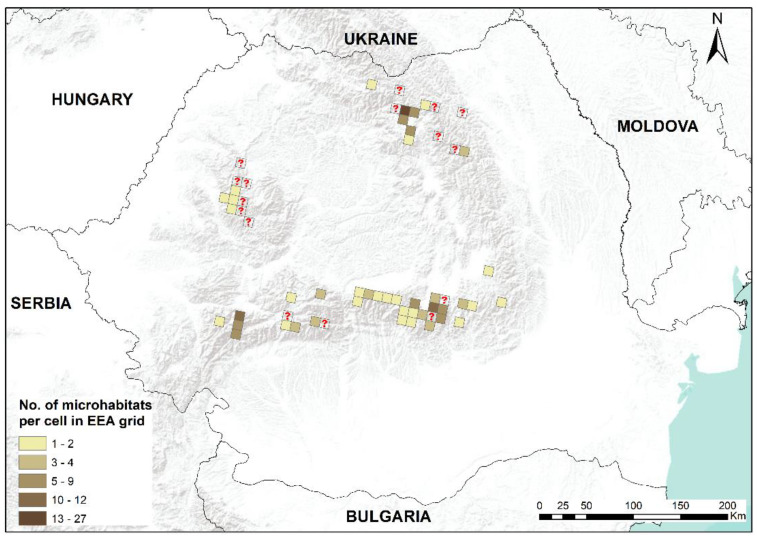
The distribution map for microhabitats of *Buxbaumia viridis* in Romania, EEA10 × 10 km grid resolution. Darker brown shades indicate an increased number of counted microhabitats per cell. Cell with a question mark represents literature data with no information on microhabitats.

**Figure 3:**
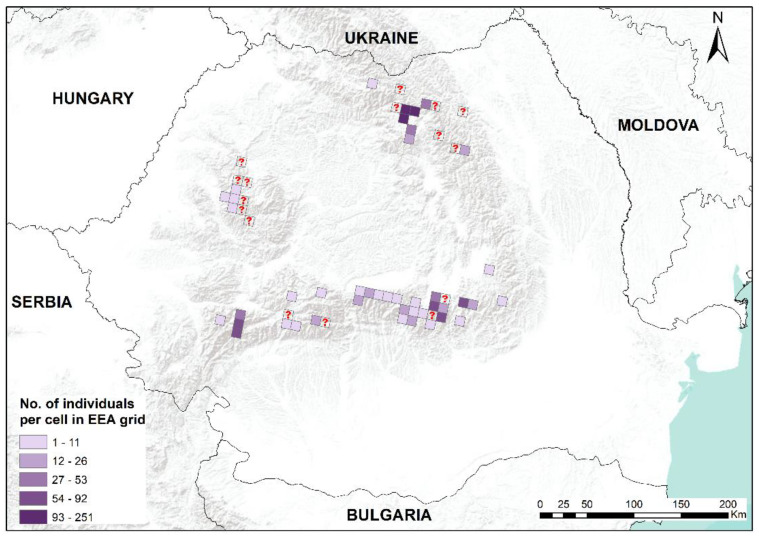
The distribution map of *Buxbaumia viridis* in Romania, with the number of individuals (sporophytes), EEA10 × 10 km grid resolution. Darker violet shades indicate an increased number of counted individuals per cell. A cell with a question mark represents literature data with no information on individuals.

**Figure 4:**
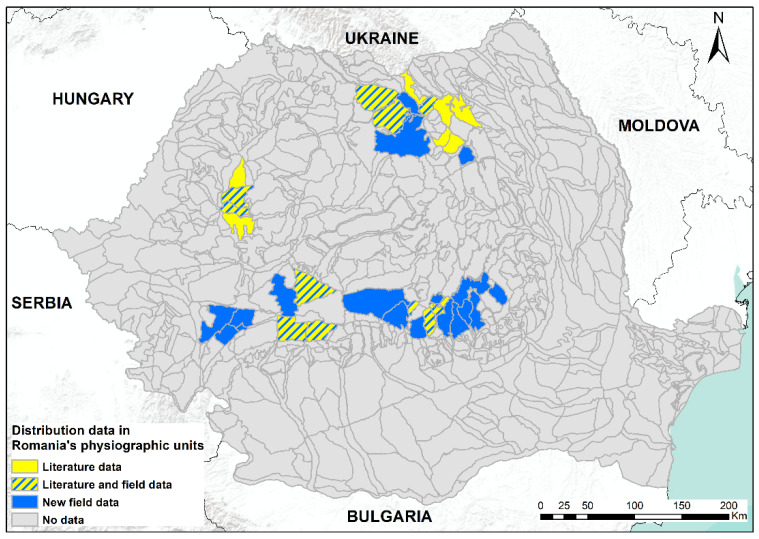
Distribution data of *Buxbaumia viridis* in Romania’s physiographic units. Blue units are new records; yellow units are based on literature data only [7,9,15,16,17,18,19,20,21,22,23,24,25,26,27,28,29,30,31,32,33,34,35,36,37,38,39]; blue/yellow hatched units are literature data confirmed in the field in the present study.

In this study, we present an updated distribution of the moss *B. viridis* in Romania, with recent field-collected data, first-time reports for 15 mountain regions and confirmations for other mountainous areas. *Buxbaumia viridis* was found in mountain massifs and lowland areas, and some mountain massifs such as Ceahlău, Vrancea, Penteleu, Siriu, Ciucaș, Grohotiș, Baiului, Postăvaru, Leaota, Făgăraș, Păpușa, Șureanu, Retezat and Țarcu represent new reports for this species. We assembled a database with 195 spatial records by combining 159 original and 36 published distribution information. We found sporophytes during snowy winter, demonstrating that it is not impossible to find them in this period [14]. All known distribution records for *B. viridis* in Romania are presented in the subsequent maps (Figure 1, Figure 2, Figure 3 and Figure 4).

**Figure 5:**
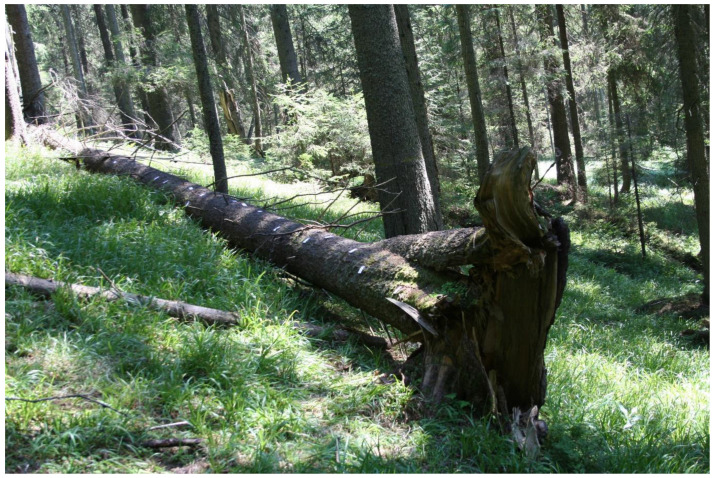
Natural spruce forest habitat with *Buxbaumia viridis* in Giumalău Mountain, Suceava County, Romania (Photo S. Ștefănuț 2007).

**Figure 6:**
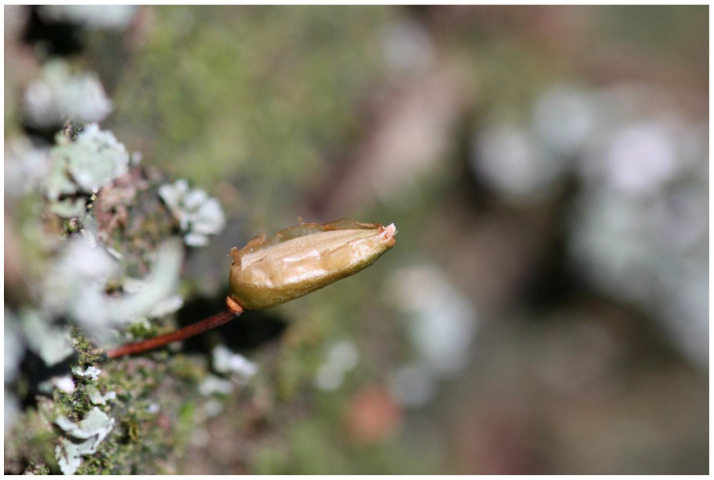
*Buxbaumia viridis* in Giumalău Mountain, Suceava County, Romania (Photo S. Ștefănuț 2007).

Until 2003, *B. viridis* was reported in these areas:

- Rodna Mountains [9,15,16];
- Suceava County, Obcina Mestecăniș, Cârlibaba Valley [17];
- Bistrița Aurie Valley [18];
- Rarău Mountain, Codrul secular Slătioara, Pârâul lui Ion, Vîiuga, Pârâului Ursului, Pârâul Ciurgău, 850–1400 m a.s.l., August 1936, *leg.* & *det.* I.T. Tarnavschi [FRE *2139*; BP *25313*; BUCA *B1486*] [7], Codrul Secular Slătioara, Pârâului Ursului, 800–1000 m a.s.l. [19];
- Munceii Rarăului, Dea, 850 m a.s.l., Valea Seacă, Poiana Mândrilă, 900–1000 m a.s.l. [7];
- Bârgău Mountains, Cucureasa Valley, 930–1230 m a.s.l. [20];
- Bistrița Mountains, Cristișor Peatbog [21];
- Stânișoara Mountains, Găinești forest chalet [22,23];
- Neamț County, Olaru Hill, 450 m a.s.l., *leg*. C. Petrescu [24];
- Piatra Mare Mountain, 1200 m a.s.l., 13 September 1962, *leg.* & *det.* L. Vajda, sub *B. indusiata* Brid. [BP *66521*], 1100 m a.s.l., 17 July 1963, *leg.* & *det.* L. Vajda, sub *B. indusiata* Brid. [BP *69257*], “Șapte Scări” Waterfall, Tamina Waterfall, 1000–1600 m a.s.l. [25];
- Brașov County, Predeal, 1200 m a.s.l. [26];
- Bucegi Mountains, Vârful cu Dor [27], Mălăiești Valley, 1400 m a.s.l., 5 September 1962, *leg.* & *det.* L. Vajda [BP *66519*] [25];
- Piatra Craiului Mountains, Gulimana (Bulimani) Valley, 1000 m a.s.l., as *B. indusiata* Brid., on rotten wood, 1100 m a.s.l., on rotten wood, 1150 m a.s.l., on rotten wood, Podul lui Călineț, western slope, 1020 m a.s.l., as *B. indusiata*, on rotten wood, Podul lui Călineț, 1050 m a.s.l., on soil [28,29], Curmătura, towards Poiana Zănoaga, 1300–1600 m a.s.l., as *B. indusiata* [15,19], Poiana Zănoaga, 1500 m a.s.l., 2 September 1962, *leg.* & *det.* L. Vajda, sub *B. indusiata* [BP *66520*], Poiana Zănoaga, 1300 m a.s.l., 2 September 1962, *leg.* & *det.* L. Vajda, sub *B. indusiata* [BP *66522*], Șpirlea Valley, *leg*. O.G. Pop, *det*. R. Wallfisch [19];
- Cibin Mountains, Păltiniș, 1100 m a.s.l. [30], Bătrâna Mountains, 1700 m a.s.l., 11 July 1963, *leg.* & *det.* L. Vajda [BP *69255*];
- Căpățânii Mountains, Repedea Valley [31];
- Parâng Mountains, Mija Stream [32];
- Apuseni Mountains, Vlădeasa Mountains, Valea Seacă, Între Munți, 19 September 1902, *leg.* & *det.* I. Györffy, sub *B. indusiata* Brid. [BP *88988*] [33]; near Lăpuș, 900–1000 m a.s.l. [34]; Drăganului Valley, Trainișu, 6 June 1963, *leg.* & *det.* L. Vajda, sub *B. indusiata* [BP *69375*] [25]; Vârciorog Valley, Arieşeni, 780 m a.s.l., 13 September 1996, *leg*. & *det*. I. Goia, Scărișoara Cave, 950 m a.s.l., 11 August 1996, *leg*. & *det*. I. Goia, Morii Valley, 950 m a.s.l., 7.08.1994, *leg*. & *det*. I. Goia, Goieștilor Valley, 1340 m a.s.l., 15.04.1994, *leg*. & *det*. I. Goia, Șaua Ursoaia, 900 m a.s.l., 12 August 1996 [35]; Galbena Valley, 780, 900 m a.s.l., 12 September 1996 [35,36]; Someșul Cald River, on decaying wood, 1998–2000 [37]; Vârciorog Valley, Arieşeni, 950 m a.s.l., 3 June 1995, *leg*. & *det*. I. Goia, between Gheţar and Ocoale, 1230 m a.s.l., 12 August 1996, *leg*. & *det*. I. Goia, Galbena Valley, Arieșeni, 870 m a.s.l., 1150 m a.s.l., 12 September1996, *leg*. & *det*. I. Goia, Iarba Rea Valley, Gârda, 980 m a.s.l., 25 August 1996, *leg*. & *det*. I. Goia [37,38];

In 2008, *B. viridis* was reported as new for Giumalău Mountain:

- Giumalău Mountain, Codrul Secular Giumalău Reserve, 47°26′45.3″ N, 25°27′49.4″ E, 1294 m a.s.l., 27 July 2007 (Figure 5 and Figure 6), 19 September 2007, 1 September 2008, *det*. S. Ștefănuț [39].

According to Romania’s report of the Habitats Directive (Directive 92/43/CEE) [1] from 2013 concerning *B. viridis* distribution in Romania, there are some dots in the western part of the Southern Carpathians.

Doubtful records for Romania:

*B. viridis* was reported from Pop Ivan Peak, Maramureș Mountains [40,41], but from the paper results, the records are from the northern part of Pop Ivan Peak, which is on the territory of Ukraine.

Also, some doubtful reports from Iezer-Păpușa Mountains [10] were excluded from this study.

**Figure 7:**
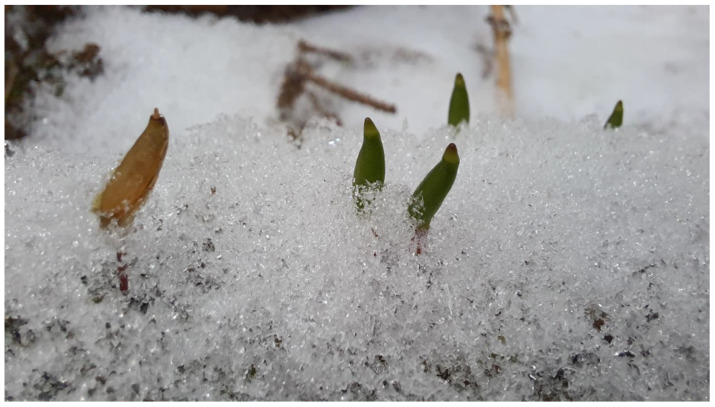
Old and young sporophytes of *Buxbaumia viridis* in winter, Poiana Stampei, Suceava County, Romania (Photo S. Ștefănuț, December 2022).

**Figure 8:**
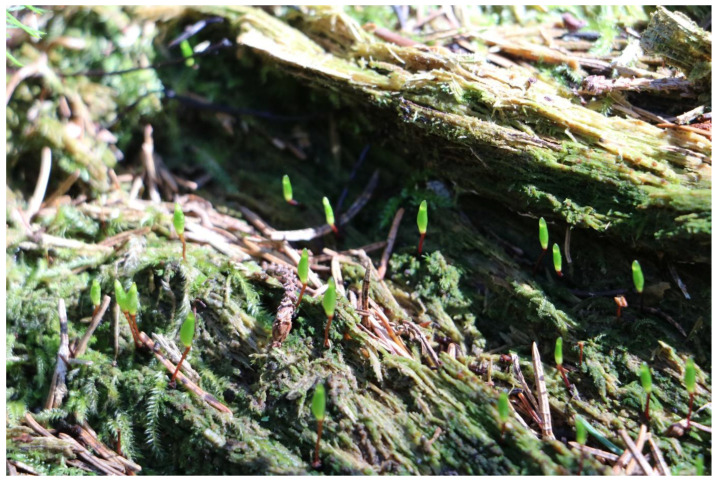
Young sporophytes of *Buxbaumia viridis* in Giumalău Mountain, Suceava County, Romania (Photo S. Ștefănuț 2018).

**Figure 9:**
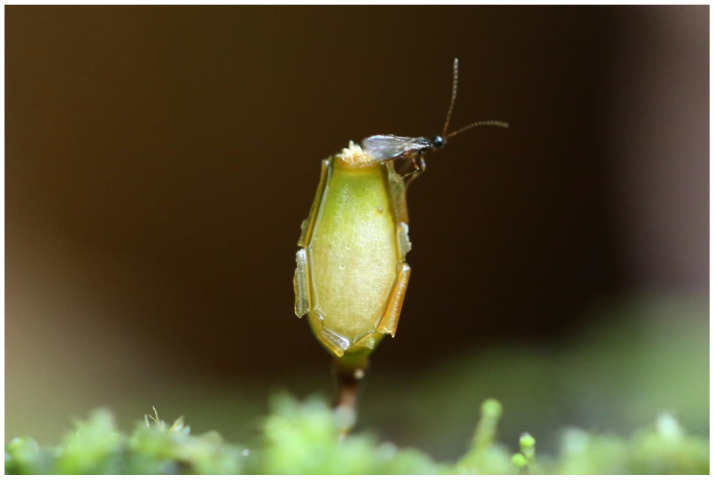
*Buxbaumia viridis* in Piatra Craiului National Park, Romania (Photo S. Ștefănuț 2019).

New records of *Buxbaumia viridis*:

- Rodna Mountains, Pietroasa, 47°36′35.3″ N, 24°39′01.3″ E, 1500 m a.s.l., 10 September 2021, *det*. G. Tamas;
- **Suceava County**, Dorna Valley, **Poiana Stampei Peatbog**, 47°18′10.86″ N, 25°08′03.84″ E, 905 m a.s.l., *det*. G. Tamas, C.-C. Bîrsan & M.C. Ion, on spruce rotten woods, 3 logs, 47°18′10.5″ N, 25°08′04.1″ E, 906 m a.s.l., 23 September 2022, *det*. S. Ștefănuț, F.-E. Helepciuc, A.-M. Moroșanu, C.-C. Bîrsan, M.-M. Ștefănuț, G. Tamas & G-R. Nicoară; on spruce rotten woods, 4 logs, 47°17′40″ N, 25°07′14″ E, 920 m a.s.l., 17 October 2022, *det*. S. Ștefănuț, C.-M. Ion, C.-C. Bîrsan & M.-M. Ștefănuț, 13 December 2022, *det*. S. Ștefănuț & A.-M. Moroșanu (Figure 7); **Bahnele Bancului 2, Bancului Valley**, on rotten wood, one log, 47°23′43.8″ N, 25°11′27.2″ E, 895 m a.s.l., 23 June 2022, *det*. S. Ștefănuț & C.-C. Bîrsan; **Teșna Valley**, on rotten wood, 4 logs, 47°21′38.3″ N, 25°06′10.8″ E, 885 m a.s.l., 24 September 2022, *det*. M.-M. Ștefănuț, A.-M. Moroșanu, F.-E. Helepciuc, C.-C. Bîrsan & S. Ștefănuț; Cucureasa Valley, on rotten woods, spruce wood: 20 logs, beech tree wood: one log, alder tree wood (*Alnus incana* (L.) Moench): one log, 47°22′18″ N, 25°05′31″ E, 848–910 m a.s.l., 24 September 2022, *det*. S. Ștefănuț, A.-M. Moroșanu, F.-E. Helepciuc, C.-C. Bîrsan & M.-M. Ștefănuț; **Oușoru Mountain**, on spruce rotten woods, 4 logs, 47°22′53″ N, 25°13′51″ E, 1070 m a.s.l., 4 logs, 47°22′31″ N, 25°14′13″ E, 944 m a.s.l., 16 October 2022, *det*. S. Ștefănuț, C.-M. Ion, C.-C. Bîrsan & M.-M. Ștefănuț;
- Giumalău Mountain, Codrul Secular Giumalău Reserve, 47°26′40.53″ N, 25°27′39.78″ E, 1240 m a.s.l., 1 September 2008, *det*. S. Ștefănuț, Codrul Secular Giumalău Reserve, 47°26′45.3″ N, 25°27′49.4″ E, 1294 m a.s.l., 19 September 2018, *det*. S. Ștefănuț, G. Tamas & C.-C. Bîrsan (Figure 8);
- **Călimani Mountains**, Haita Valley, on rotten woods, 4 logs, 47°11′21″ N, 25°15′04″ E, 1096–1102 m a.s.l., 4 logs, 47°10′15″ N, 25°14′44″ E, 1132–1170 m a.s.l., 25 September 2022, *det*. S. Ștefănuț, A.-M. Moroșanu, F.-E. Helepciuc, C.-C. Bîrsan, M.-M. Ștefănuț & G. Tamas; Călimani National Park, on rotten wood, one log, 47°06′30.9″ N, 25°14′39.8″ E, 1297 m a.s.l., one logs, 47°07′44.4″ N, 25°14′56.7″ E, 1545 m a.s.l., 14 October 2022, *det*. S. Ștefănuț, C.-M. Ion, C.-C. Bîrsan, M.-M. Ștefănuț & G. Tamas;
- **Ceahlău Mountain**, Ceahlău Peak, 46°59′39.1″ N, 26°13′30.2″ E, 948 m a.s.l., Bucur Valley, 46°59′45.5″ N, 25°55′52.3″ E, 948 m a.s.l., Durău Valley, 46°59′51.8″ N, 25°56′06.7″ E, 922 m a.s.l., 11 August 2021, *det*. G. Tamas & C.-C. Bîrsan;
- **Vrancea Mountains**, Covasna Valley, 45°49′32.3″ N, 26°13′30.2″ E, 819 m a.s.l., 29 May 2021, *det*. G. Tamas;
- **Penteleu Mountain**, Brebu Valley, on rotten wood, two logs, 45°35′09.9″ N, 26°27′42.7″ E, 1105 m a.s.l., 11 July 2022, *det*. C.-C. Bîrsan & S. Ștefănuț;
- **Siriu Mountain**, Siriu Mare Valley, 45°29′34.3″ N, 26°05′11.8″ E, 975 m a.s.l., on rotten alder wood (*Alnus incana* (L.) Moench), one log, 7 October 2021, *det*. S. Ștefănuț, C.-C. Bîrsan, G. Tamas & G.-R. Nicoară;
- **Ciucaș Mountains**, Prundului Valley, on rotten wood, two logs, 45°32′32.0″ N, 25°55′26.5″ E, 1014 m a.s.l., 45°32′30.6″ N, 25°55′26.0″ E, 1066 m a.s.l., Strâmbu Valley, on rotten wood, two logs, 45°32′15.9″ N, 25°57′59.0″ E, 1209 m a.s.l., 45°32′15.4″ N, 25°57′54.2″ E, 1246 m a.s.l., 9 September 2020, *det*. S. Ștefănuț, C.-C. Bîrsan & G.-R. Nicoară;
- **Grohotiș Mountains**, Bobului Valley, on rotten wood, one log, 45°24′12.0″ N, 25°53′14.2″ E, 1200 m a.s.l., 1 July 2020, *det*. S. Ștefănuț, C.-C. Bîrsan & G. Tamas;
- Piatra Mare Mountain, 45°32′08.3″ N, 25°35′27.8″ E, 869 m a.s.l., 45°32′08.2″ N, 25°35′28.0″ E, 871 m a.s.l., 45°32′06.2″ N, 25°35′34.6″ E, 905 m a.s.l., 45°32′05.0″ N, 25°35′37.0″ E, 912 m a.s.l., 45°32′05.1″ N, 25°35′37.9″ E, 914 m a.s.l., 45°32′04.9″ N, 25°35′37.6″ E, 916 m a.s.l., 17 December 2020, *det*. S. Ștefănuț, C.-C. Bîrsan & G. Tamas.
- Brașov County, Predeal, 45°31′26.5″ N, 25°33′08.6″ E, 1065 m a.s.l., 17 December 2020, *det*. C.-C. Bîrsan, G. Tamas & S. Ștefănuț;
- **Postăvaru Mountain**, Lamba Mare Valley, on rotten wood, one log, 45°34′47.3″ N, 25°34′30.9″ E, 1082 m a.s.l., one log, 45°34′46.9″ N, 25°34′28.4″ E, 1097 m a.s.l., 29 July 2020, *det*. S. Ștefănuț, G. Tamas & C.-C. Bîrsan; Vanga Mare Valley, 45°34′05.0″ N, 25°33′20.0″ E, 1608 m a.s.l., 7 June 2021, *det*. S. Ștefănuț, C.-C. Bîrsan G. Tamas & G.-R. Nicoară;
- **Baiului Mountains**, Rea Valley, on rotten wood, one log, 45°21′52.9″ N, 25°36′00.4″ E, 1076 m a.s.l., two logs, 45°21′53.1″ N, 25°35′59.8″ E, 1077 m a.s.l., 45°21′54.2″ N, 25°36′00.1″ E, 1087 m a.s.l., two logs, 45°21′54.8″ N, 25°36′00.3″ E, 1089 m a.s.l., 3 September 2020, *det*. C.-C. Bîrsan, G. Tamas, G.-R. Nicoară & S. Ștefănuț;
- Bucegi Mountains, Mălăiești Valley, on rotten woods, 45°29′18.9″ N, 25°28′27.0″ E, 973 m a.s.l., 45°29′19.2″ N, 25°28′26.6″ E, 990 m a.s.l., 45°29′18.0″ N, 25°28′26.7″ E, 999 m a.s.l., 45°29′03.8″ N, 25°28′30.8″ E, 1051 m a.s.l., 45°29′03.7″ N, 25°28′31.0″ E, 1058 m a.s.l., 45°29′03.5″ N, 25°28′30.7″ E, 1060 m a.s.l., 45°29′02.5″ N, 25°28′29.6″ E, 1063 m a.s.l., 45°29′00.5″ N, 25°28′29.1″ E, 1067 m a.s.l., 45°29′00.3″ N, 25°28′28.6″ E, 1067 m a.s.l., 45°28′59.0″ N, 25°28′26.8″ E, 1076 m a.s.l., 17 June 2020, *det*. S. Ștefănuț, G. Tamas & C.-C. Bîrsan; **Guțanu Valley**, one log, 45°25′05.2″ N, 25°23′50.3″ E, 1573 m a.s.l., one log, 45°25′06″ N, 25°23′50.2″ E, 1573 m a.s.l., **Culmea Grohotișului**, one log, 45°24′12.5″ N, 25°23′04.1″ E, 1520 m a.s.l., 10 October 2020, *det*. G. Tamas; **Cota 1400**, one log, 45°21′14.6″ N, 25°30′55.5″ E, 1440 m a.s.l., 12 August 2022, *det*. S. Ștefănuț, M.-M. Ștefănuț; **Peleș Valley**, one log, 45°21′41.5″ N, 25°31′44.2″ E, 1044 m a.s.l., 13 August 2022, *det*. S. Ștefănuț, M.-M. Ștefănuț; near **Cuibul Dorului chalet**, two logs, 45°19′12.7″ N, 25°30′51.5″ E, 1427 m a.s.l., 6 Sptember 2022, *det*. S. Ștefănuț, G. Tamas, Nicoară G.-R. & C.-C. Bîrsan;
- **Leaota Mountain**, Crovului Gorges, one log, 45°24′04.0″ N, 25°15′38.5″ E, 1012 m a.s.l., 30 October 2021, *det*. G.-R. Nicoară, one log, 45°23′31.9″ N, 25°16′36.9″ E, 1295 m a.s.l., 24 July 2022, *det*. G. Tamas;
- Piatra Craiului Mountains, near Garofița Pietrei Craiului chalet, beech forest, on soil, 45°30′45.74″ N, 25°10′23.57″ E, 1120 m a.s.l., Piscul Rece, 45°30′39.77″ N, 25°11′10.04″ E, 1350 m a.s.l., 9 July 2003, *det*. S. Ștefănuț; Șpirlea Valley, on rotten wood, one log, 45°32′25.80″ N, 25°11′38.40″ E, 1077 m a.s.l., 25 June 2019, *det*. S. Ștefănuț, G. Tamas, C-C. Bîrsan & M.C. Ion; Vlădușca Valley, on rotten wood, 3 logs, 45°32′52.20″ N, 25°11′45.30″ E, 1029 m a.s.l., on rotten wood, one log, 45°32′50.90″ N, 25°11′49.50″ E, 1040 m a.s.l., 16 July 2019, *det*. S. Ștefănuț, G. Tamas, C.-C. Bîrsan & M.-M. Ștefănuț (Figure 9); Podurilor Valley, 45°32′36.00″ N, 25°12′30.00″ E, 1360 m a.s.l., 17 July 2019, *det*. S. Ștefănuț, G. Tamas, C.-C. Bîrsan & M.-M. Ștefănuț; Padina Lancii Valley, 45°30′39.3″ N, 25°11′23.9″ E, 1417 m a.s.l., 27 July 2022, *det*. G. Tamas;
- **Făgăraș Mountains**, below Bârcaciu chalet, 45°36′58.30″ N, 24°28′45.00″ E, 1226 m a.s.l., 26 August 2015, *det*. S. Ștefănuț; Podragu Valley, near Turnuri chalet, 45°37′35.25″ N, 24°40′34.20″ E, 1500 m a.s.l., 29 August 2017, Arpașul Mare Valley, 45°38′32.91″ N, 24°40′19.88″ E, 1140 m a.s.l., 29 August 2017, *det*. S. Ștefănuț & C.-C. Bîrsan; Breaza Valley, 45°38′17.54″ N, 24°52′28.74″ E, 1103 m a.s.l., 25 July 2019, *det*. S. Ștefănuț, G. Tamas & C.-C. Bîrsan; Viștișoara Valley, 45°38′58.9″ N, 24°45′56.0″ E, 1120 m a.s.l., 19 August 2020, *det*. S. Ștefănuț, G. Tamas, M. Vladimirescu & C.-C. Bîrsan; Boia Valley, 45°32′37″ N, 24°26′58″ E, 1610 m a.s.l., 28 September 2020, *det*. G.-R. Nicoară; Șerbota Valley, 45°36′35.8″ N, 23°57′14.2″ E, 1163 m a.s.l., 23 May 2021, *det*. S. Ștefănuț, C.-C. Bîrsan, A.-M. Moroșanu, F.-E. Helepciuc & G. Tamas; Arpășel Valley, 45°39′19.6″ N, 24°37′34.4″ E, 921 m a.s.l., 9 September 2021, *det*. S. Ștefănuț, C.-C. Bîrsan, A.-M. Moroșanu & F.-E. Helepciuc; Dâmbovița Valley, 45°34′14.4″ N, 25°03′36.9″ E, 1163 m a.s.l., 11 November 2022, *det*. S. Ștefănuț;
- **Iezer-Păpușa Mountains**, Păpușa Mountain, Cuca Valley, on rotten wood, one log, 45°28′23.9″ N, 25°02′40.1″ E, 1180 m a.s.l., 8 July 2020, *det*. C.-C. Bîrsan, G. Tamas & S. Ștefănuț; Valea Rea, 45°26′31.6″ N, 25°03′23″ E, 1080 m a.s.l., 31 October 2021, *det*. G. Tamas;
- Cindrel Mountains, Steaza Valley, 45°40′07.4″ N, 23°57′17.4″ E, 1122 m a.s.l., 45°40′07.3″ N, 24°31′14.2″ E, 1131 m a.s.l., Valea lui Andrei, 45°39′16.1″ N, 23°57′11.8″ E, 1263 m a.s.l., 22 May 2021, *det*. S. Ștefănuț, C.-C. Bîrsan, A.-M. Moroșanu, F.-E. Helepciuc & G. Tamas;
- **Șureanu Mountain**, Cugir Valley, 45°35′43.6″ N, 23°30′52.3″ E, 1484 m a.s.l., 45°35′42.3″ N, 23°30′50.6″ E, 1486 m a.s.l., 29 July 2021, *det*. G. Tamas, S. Ștefănuț, C.-C. Bîrsan & G.-R. Nicoară;
- Căpățânii Mountains, Repedea Valley, 45°19′59.0″ N, 23°52′27.1″ E, 1245 m a.s.l., 45°19′58.1″ N, 23°52′25.2″ E, 1279 m a.s.l., 26 July 2021, *det*. S. Ștefănuț, C.-C. Bîrsan, G. Tamas & G.-R. Nicoară;
- Parâng Mountains, Lotru Valley, on rotten woods, two logs, 45°21′52.21″ N, 23°37′16.45″ E, 1637 m a.s.l., one log, 45°21′55.26″ N, 23°37′14.20″ E, 1626 m a.s.l., 29 August 2020, *det*. G. Tamas & G.-R. Nicoară; Roșiile Valley, 45°21′52.21” N, 23°37′16.45″ E, 1637 m a.s.l., one log, 45°22′10.24″ N, 23°33′53.51″ E, 1510 m a.s.l., 25 August 2022, *det*. G. Tamas;
- **Retezat Mountains**, Pietrele Valley, on rotten woods, 45°24′20.5″ N, 22°53′11.7″ E, 1432 m a.s.l., 45°24′25.4″ N, 22°53′17.0″ E, 1451 m a.s.l., Galeș Valley, on rotten woods, 45°24′19.6″ N, 22°53′28.0″ E, 1461 m a.s.l., 45°24′19.7″ N, 22°53′28.3″ E, 1465 m a.s.l., 45°24′17.2″ N, 22°53′28.7″ E, 1480 m a.s.l., 45°24′17.3″ N, 22°53′29.0″ E, 1479 m a.s.l., 45°24′16.9″ N, 45°24′16.9″ N, 1481 m a.s.l., 45°24′15.1″ N, 22°53′29.6″ E, 1487 m a.s.l., 45°24′07.4″ N, 22°53′35.3″ E, 1509 m a.s.l., 45°24′05.2″ N, 22°53′39.7″ E, 1527 m a.s.l., 45°24′09.6″ N, 22°53′45.2″ E, 1562 m a.s.l., on soil, 45°24′05.5″ N, 22°53′38.8″ E, 1521 m a.s.l., 23 July 2020, *det*. S. Ștefănuț, C.-C. Bîrsan, G. Tamas & G.-R. Nicoară; Scorota Valley, on rotten woods, 45°16′51.0″ N, 22°53′42.5″ E, 1168 m a.s.l., 45°16′55.3″ N, 22°53′30.4″ E, 1206 m a.s.l., 45°16′55.1″ N, 22°53′30.4″ E, 1210 m a.s.l., 45°16′56.0″ N, 22°53′28.8″ E, 1215 m a.s.l., 45°17′00.1″ N, 22°53′29.3″ E, 1227 m a.s.l., 45°17′05.6″ N, 22°53′34.1″ E, 1240 m a.s.l., 45°17′06.0″ N, 22°53′33.8″ E, 1241 m a.s.l., 45°17′06.9″ N, 22°53′35.7″ E, 1245 m a.s.l., Iarului Valley, 45°16′05.3″ N, 22°51′18.1″ E, 1332 m a.s.l., 45°16′07.3″ N, 22°51′12.9″ E, 1357 m a.s.l., 45°16′07.3″ N, 22°51′12.1″ E, 1359 m a.s.l., 45°16′09.4″ N, 22°51′10.7″ E, 1369 m a.s.l., 45°16′12.6″ N, 22°51′10.8″ E, 1374 m a.s.l., 45°16′12.9″ N, 22°51′10.8″ E, 1382 m a.s.l., 45°16′13.1″ N, 22°51′10.4″ E, 1381 m a.s.l., 13 August 2020, *det*. C.-C. Bîrsan;
- **Țarcu Mountains**, Mătania Valley, on rotten woods, two logs, 45°18′18.26″ N, 22°39′16.1″ E, 1241 m a.s.l., 45°18′17.6″ N, 22°39′15.0″ E, 1257 m a.s.l., 15 September 2020, *det*. S. Ștefănuț, G.-R. Nicoară, C.-C. Bîrsan & G. Tamas;
- Bihor County, Buciniș Valley, 46°26′31.2” N, 22°44′20.8″ E, 1227 m a.s.l., 18 July 2021, *det*. G. Tamas;
- Apuseni Mountains, Padiș, 46°34′33.99″ N, 22°42′12.31″ E, 1110 m a.s.l., 3 September 2007, *det*. S. Ștefănuț; Peștera Coiba Mare, 46°32′14.6″ N, 22°46′40.7″ E, 1100 m a.s.l., 19 July 2021, *det*. G. Tamas; Ghețarul Focul Viu, 46°34′30.01″ N, 22°40′54.2″ E, 1162 m a.s.l., 20 July 2021, *det*. G. Tamas; Avenul Bortig, 46°33′34.3″ N, 22°41′50.5″ E, 1164 m a.s.l., 20 July 2021, *det*. G. Tamas.

### 2.2. Habitat Suitability

The distribution modeling displayed good metric performance with mean AUC values between 0.81 and 0.89 and mean TSS values in the range of 0.58 to 0.67 (Table 1). ROC plots also displayed good performances by all modeling algorithms, with AUC values > 0.8 (Figures S1–S3). The highest contributing variables were elevation (16%), precipitation of driest quarter (12.9%), and mean temperature of coldest quarter (11.4%), followed by precipitation of warmest quarter (8.1%), percentage of tree cover (3.6%), heat load index (3.3%), leaf area index (3%) and Euclidean distance to the nearest river with the lowest contribution in constructing the model (1.2%) (Table 2, Figure S4). The response curves show that the species’ range encompasses mainly high-altitude areas, generally located between 1000 and 1500 m, with high levels of precipitation during the driest quarter of the year (above 150 mm), low mean temperatures during the coldest quarter (below −5 °C) and high precipitations during the warmest quarter (about 300 mm) (Figure S4); increased tree cover, slopes with low heat load index, moderate leaf area index are also important in defining the environmental space for the green-shield moss (Figure S5).

**Figure 10:**
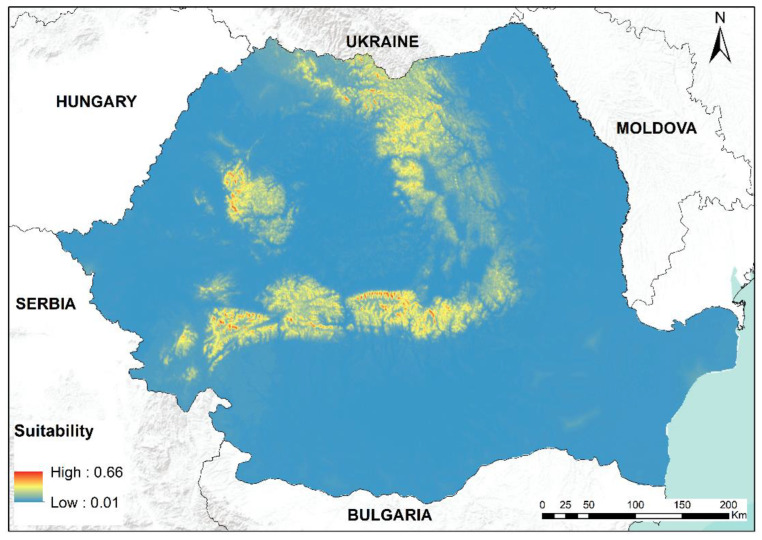
The ensemble habitat suitability model for *Buxbaumia viridis*. Blue shades indicate low suitability, while red shades represent high suitability.

With regard to the known distribution of *Buxbaumia viridis*, the present model of habitat suitability showed remarkable congruence. The ensemble model highlighted a high density of suitable habitats for *B. viridis* in most of the mountain massifs where the species was found, such as Retezat, Bucegi, Făgăraș, Piatra Craiului and the western part of Apuseni Mountains (Figure 10). Furthermore, wide areas of highly suitable habitats for this moss have been identified in Gutâi, Țibles and Maramureș mountains in the north, but also Gurghiu and Semenic mountains. The central part of Eastern Romanian Carpathians has mostly isolated areas of suitable low to medium habitats, as do the Poiana Ruscă Mountains and the eastern part of the Apuseni Mountains. Out of all 167 physiographic units identified as holding some potential areas for the species’ range by the threshold model, 129 have no known occurrence records.

The field surveys carried out resulted in 218 microhabitats colonized by *B. viridis* at the national level. From these, 172 microhabitats have been recorded in this study, with a total of 1534 sporophytes. In Romania, *B. viridis* is characterized by the fact that it colonizes spruce deadwood [3], as opposed to its occurrence on soil in Hungary [2]. Our field activities identified 162 microhabitats (>94%) belonging to coniferous deadwood, mainly *Picea abies* (L.) H. Karst., five microhabitats were beech deadwood (*Fagus sylvatica* L.), two microhabitats were white alder deadwood (*Alnus incana* (L.) Moench) and three microhabitats were on soil.

### 2.3. Population and Conservation Status

Considering that the Area of Occupancy (AOO) according to our study is 268 km^2^, representing approximately 1% of the total potential distribution range of the species, we can conclude that the total population of *Buxbaumia viridis* in Romania is over 150,000 sporophytes distributed in more than 17,000 microhabitats.

We propose that the conservation status of *Buxbaumia viridis* in Romania should be changed from Endangered–EN A3c; C1 [5,12] to Vulnerable–VU A3c.

## 3. Discussion

Our research showed, as we expected that in places where the natural and mature spruce forest habitats are present, the probability that *Buxbaumia viridis* is present is very high. Moreover, the area covered by spruce sums up to 1.37 million hectares, as estimated in the latest National Forest Inventory [42]; hence a large area with potential habitat in Romania is still uncovered by research.

Although it is an ephemeral species, *B. viridis* appears from August–September and matures the following year, sporulating from May–July [43]; populations can be found years in a row in the same place. For example, in the Giumalau Massif, the species was identified on a spruce log in 2007 and 2008 [39], and in 2018 it was found on the same log, in the same area of the log, at the base, so after a period of 12 generations.

The compiled distribution map, overlaid with the borders of all the sites of Community importance (SCI, as part of the NATURA 2000 network), revealed that 20 more areas have no protection at all, and these will be part of the new NATURA 2000 site proposals.

According to the results from the ensemble modeling, elevation, precipitation of the driest quarter, and mean temperature of the coldest quarter serve as the best indicators of *Buxbaumia viridis* occurrence. We can observe that the favored high-altitude areas typically correlate with the altitudinal range of mixed and spruce forests; hence there might be a dependence on habitat type, also supported by other studies [44,45]. Using maximum entropy modeling, Číhal [44] also argues that the found dependency of *B. viridis* to habitat type is most likely mostly connected to the species’ requirement for a sufficient quantity of decaying wood [46]. However, when focusing on desiccation, Kropik [47] found that this variable outperforms decaying wood in terms of predicting power for explaining the occurrence of this moss in Austria. This result indicates that climate can have a more substantial effect on the distribution. Our data suggest the same, as precipitation of the driest quarter and mean temperature of the coldest quarter accompanied by precipitation of the warmest quarter are the important consequent variables after elevation. Increased precipitation (in the driest and warmest areas) has a direct influence on avoiding the desiccation of not just sporophytes [45,46] but also of the less tolerant spores [48]. The reason for the species’ dependency on lower average winter temperatures is uncertain, as also found and stated by Číhal [44]. Only 3 °C of winter warming in several experimental plots in the UK caused a cover increase in certain moss species and a decrease in cover in other species, under a general decrease of bryophytes species richness. Little is known about bryophytes’ spore dormancy [49] or spore development that could explain this dependency. Other causes could be indirect. 

In the Pyrenees, the effects of organisms grazing on sporophyte were observed in spring, sometimes with slugs from the *Arion* genus being responsible [50]. However, slugs can be active even in winter, and the activity of some species increases when the soil temperature is above 10 °C [51]; decaying wood is also known to act like temperature microhabitats refuges for soil invertebrates. For example, adults of *Arion distinctus* Mabille, 1898 are thought to survive in winter and even lay eggs throughout these months until spring [52]. Nevertheless, despite their freeze tolerance, slug species from the same genus do not survive below −3 °C [53]. Therefore, we could argue that low mean temperatures below −5 °C might reduce the slugs’ survival and density, hence the grazing intensity on *B. viridis* sporophytes. Both spore dormancy/development and bryophagy effect over this moss during winter, in correlation with low temperatures, are worth further exploring.

The next three variables in order of importance in producing the model are the percentage of tree cover, heat load index, and leaf area index. Slopes with low heat load index also indicate shadier and more humid forest floor conditions, which is in agreement with findings about “Northness” in other papers [45,54], but with a finer prediction due to the calculation method [55]. Spitale and Mair [54] also found that this moss species prefers closed-canopy forests. Canopy closure, percentage tree cover and leaf area index are structural variables describing the canopy. The canopy has an essential impact on understory composition and species cover in a forest [56] by influencing light, humidity and even soil pH. Our data showed that this moss is most likely found in areas with increased tree cover but a moderate leaf area index. In our field experience, coniferous forests with higher leaf area index tend to accumulate a thicker layer of needles that seemed to hinder gametophyte development.

As we suspected, the model for habitat suitability detected physiographic units (mountains and lowland areas) within the species range, some with high suitability, such as Gurghiu and Semenic. These areas, as well as the northern part of the Romanian Carpathians (Maramureș mountains), should be the first target in future field studies. The record from Stânișoara Mountains, Găinești forest chalet is the only one that is situated outside the species’ range. With new field data, especially from the central outer part of the Eastern Romanian Carpathians, the model results could be improved, but it is also worth confirming the record again, as the last mention was from four decades ago [23];

Previous research indicated that deadwood amount is an important variable influencing *B. viridis* distribution predictions [46,47,54], which could improve distribution modeling. However, spatial databases and statistics regarding forest deadwood were only recently published, with a low resolution of 16 km, at the European level and did not cover Romanian territory [57]. Furthermore, some anthropogenic-related disturbances like air pollution were found to have negligible influence on this moss distribution [44]. Other categorial predictors like land use (Corine Land Cover) or forest management methods are sometimes found significant [44,45], but these also determine deadwood volume, at least in some forest types [58]. In addition, land use data are usually highly correlated with other environmental predictors, such as the climatic envelope. Moreover, categorical predictors are difficult to include in species distribution modeling since not all algorithms successfully manage them.

The new reports of *B. viridis* from areas not currently protected, such as Capățânii, Baiului, Grohotişului and Oușor Mountains, offer support for the designation of new Natura 2000 sites, as well as the opportunity to expand the limits of existing Natura 2000 sites of community importance, especially ROSCI0101 Larion, ROSCI0019 Călimani-Gurghiu, ROSCI0208 Putna-Vrancea, ROSCI0190 Penteleu, ROSCI0229 Siriu and ROSCI0207 Postăvarul.

## 4. Methods

### 4.1. Occurrence Records

An extensive field survey for *B. viridis* was conducted by the authors in South-Eastern Carpathians in the last 20 years; the following data were collected for each occurrence: geographic coordinates (decimal degrees) and elevation using a GPS receiver, the number of microhabitats (logs or 1 m^2^ area for soil) and number of sporophytes in all identified presence locations. A critical review of scientific publications was carried out, and distribution data were registered in a database. When no coordinates were provided, toponyms were georeferenced using Google Earth Pro version 7.3.6.9345, Google Maps version 2022, and military survey maps for Romania with an assumed error of less than 1 km. Distribution maps were created using the collected spatial data in ArcGIS 10.7.1 [59].

### 4.2. Model Preparation and Procedure

We used both original and published distribution records for the green-shield moss (*B. viridis*); before modeling, we rarefied the occurrence dataset using SDMToolbox v2.5 add-on [60] in ArcGIS 10.7.1 and a distance of 1 km to match the spatial resolution of the input predictors; the resulting dataset used for modeling comprised 95 occurrence records.

Regarding environmental predictors, we started with a package that contained 12 variables, describing climatic, ecological and geomorphological conditions in the area where the species is distributed (Table 2). For the bioclimatic predictor, we used the second-generation baseline (v2.1) [61], representative of the 1970–2000 period. Heat Load Index (HLI) was calculated using the Geomorphometry and Gradient Metric Toolbox v2.0–0 [62]. All variables were used at a resolution of 30” (~1 km).

We first split the environmental rasters into two sets of predictors: (1) one set for training, clipped to the minimum convex polygon enclosing the input features (convex hull) plus a generic buffer of 10 km, and (2) a second set for predictors, clipped to the border of Romania plus a generic buffer of 10 km. This phase was carried out in ArcGIS 10.7.1 using the SDMToolbox v2.5 add-on.

Before the modeling phase, multicollinearity among variables was tested using the Variance Inflation Factor (VIF) implemented in the usdm package [63] in R version 4.2.1 [64] and excluded variables with VIF > 10 [65]. The final list of predictors used is presented in Table 2.

We employed ensemble modeling in R using the sdm package [66] to predict habitat suitability for green-shield moss (*B. viridis*) in Romania. Models were generated using the training predictors and six methods, GLM (Generalized Linear Models), RF (Random Forests), Maxent, BRT (Boosted Regression Trees), MARS (Multivariate Adaptive Regression Spline) and SVM (Support Vector Machine), and then projected onto the entire environmental space (as defined above). Settings included 10.000 random background points, 5-fold cross-validation with 10 repetitions, and a test percentage of 30%.

In total, 300 models were generated and evaluated based on AUC and TSS. The ensemble was generated from models with AUC values greater than the mean plus half a standard deviation or models with TSS values greater than the mean plus half a standard deviation [67]. A threshold model was also created based on the TSS cut-off value.

### 4.3. Conservation Status Assessment

Conservation status was reevaluated based on the new distribution records from the current article and the IUCN methodology. AOO was calculated in ArcGIS 10.7.1 using a 2 km square grid, as recommended in the IUCN Red List Categories and Criteria [68].

## 5. Conclusions

This study can be used to designate new NATURA 2000 sites or enlarge the areas of the actual sites for the *B. viridis* species in Romania, which would contribute to the fulfillment of the target assumed by The European Commission through the EU Biodiversity Strategy for 2030: Bringing nature back into our lives in 2020, to establish a protected area for at least 30% of land in Europe [69].

## Figures and Tables

**Table 1:** Mean performance statistics for each modeling method.

Method	MeanAUC	MeanTSS
GLM	0.81	0.58
RF	0.89	0.67
Maxent	0.88	0.65
BRT	0.88	0.66
MARS	0.86	0.62
SVM	0.82	0.6

**Table 2:** List of environmental variables used for modeling.

Code	Name	Source	Relative Importance (%)
bio9 *	Mean Temperature of Driest Quarter	www.worldclim.org (accessed on 16 November 2022)	-
bio10 *	Mean Temperature of Warmest Quarter	www.worldclim.org (accessed on 16 November 2022)	-
bio11	Mean Temperature of Coldest Quarter	www.worldclim.org (accessed on 16 November 2022)	11.4
bio12 *	Annual Precipitation	www.worldclim.org (accessed on 16 November 2022)	-
bio16 *	Precipitation of Wettest Quarter	www.worldclim.org (accessed on 16 November 2022)	-
bio17	Precipitation of Driest Quarter	www.worldclim.org (accessed on 16 November 2022)	12.9
bio18	Precipitation of Warmest Quarter	www.worldclim.org (accessed on 16 November 2022)	8.1
dem	Elevation	www.worldclim.org (accessed on 16 November 2022)	16
dist_river	Euclidean distance to nearest river	Calculated in ArcGIS	1.2
HLI	Heat Load Index	Calculated in ArcGIS	3.3
LAI	Leaf Area Index	https://land.copernicus.eu (accessed on 16 November 2022)	3
tree_cover	Percentage tree cover	https://land.copernicus.eu (accessed on 16 November 2022)	3.6

* excluded from the modeling phase based on VIF.

## Data Availability

All data on *Buxbaumia viridis* distribution are presented in this study and are available in the main article.

## Supplementary Materials

The following supporting information can be downloaded at: https://www.mdpi.com/article/10.3390/plants12030473/s1, Figure S1: ROC-AUC curves for the GLM and RF models; Figure S2: ROC-AUC curves for the Maxent and BRT models; Figure S3: ROC-AUC curves for the MARS and SVM models; Figure S4: Relative variable importance for all models across all modeling methods (SD bars included); Figure S5: The response curves for all eight predictors

## References

[1] Directive Habitats (1992). Council Directive 92/43/EEC of 21 May 1992 on the conservation of natural habitats and of wild fauna and flora. Off. J. Eur. Communities.

[2] Deme J., Erzberger P., Kovács D., Tóth I.Z., Csiky J. (2020). *Buxbaumia viridis* (Moug. ex Lam. & DC.) Brid. ex Moug. & Nestl. in Hungary predominantly terricolous and found in managed forests. Cryptogam. Bryol..

[3] Goia I., Gafta D. (2019). Beech versus spruce deadwood as forest microhabitat: Does it make any difference to bryophytes?. Plant Biosyst. Int. J. Deal. Asp. Plant Biol..

[4] Holá E., Vrba J., Linhartová R., Novozámská E., Zmrhalová M., Plasek V., Kucera J. (2014). Thirteen years on the hunt for Buxbaumia viridis in the Czech Republic: Still on the tip of the iceberg?. Acta Soc. Bot. Pol..

[5] Hodgetts N.G., Lockhart N. (2020). Checklist and country status of European bryophytes–update 2020. Irish Wildlife Manuals, No. 123. National Parks and Wildlife Service.

[6] (2019). WWF Calls for A Public Debate on the Results of the Romanian National Forest Inventory. https://wwf.panda.org/wwf_news/?357022/debated-nfi.

[7] Tarnavschi I. (1936). Beitrag zur Oekologie und Phytosoziologie der Buxbaumia indusiata Bridel sowie zur Verbreitung von *Buxbaumia aphylla* L. und Buxbaumia indusiata Brid. Bul. Fac. De Ştiinţe Din Cernăuţi.

[8] Papp C. (1967). Briofitele din Republica Socialistă România (determinator). Anal. Şti. Univ. "Al.I. Cuza" Iaşi–Biol. Monogr..

[9] Plămadă E., Dumitru C. (1998). Flora Briologică a României. Clasa Musci 1.

[10] Mohan G. (1998). Catalogul briofitelor din România. Acta Botanica Horti Bucurestiensis.

[11] Mihăilescu S., Anastasiu P., Popescu A. (2015). Ghidul de Monitorizare a Speciilor de Plante de Interes Comunitar din România.

[12] Stefanut S., Goia I. (2012). Checklist and Red List of Bryophytes of Romania. Nova Hedwig..

[13] Hodgetts N.G. (2015). Checklist and Country Status of European Bryophytes–towards a New Red List for Europe: National Parks and Wildlife Service.

[14] Brewczyński P., Grałek K., Bilański P. (2021). Occurrence of the Green Shield-Moss Buxbaumia viridis (Moug.) Brid. in the Bieszczady Mountains of Poland. Forests.

[15] Pax F. (1898). Grundzüge der Pflanzenverbreitung in den Karpathen.

[16] Ştefănuț S. (2010). The Bryophytes of Rodna Mountains National Park (Transylvania-Maramureş, Romania). Transylv. Rev. Syst. Ecol. Res..

[17] Ştefureac T., Pascal P. (1970). Contribuţion à la bryoflore de la Bukovine (Roumanie). Rev. Roum. Biol. Série Bot..

[18] Pascal P., Mititelu D. (1971). Contribuţie la studiul vegetaţiei din bazinul Bistriţei Aurii (Jud. Suceava). Comunic. Şti. Inst. Pedagog..

[19] Ştefureac T. (1941). Cercetări sinecologice şi sociologice asupra bryophytelor din Codrul Secular Slătioara (Bucovina). An. Acad. Române. Mem. Secţiunii Ştiinţifice..

[20] Ştefureac T., Mihai G., Pascal P. (1976). Cercetări briologice în rezervaţia forestieră Cucureasa (Vatra Dornei). St. Cerc. Biol. Ser. Biol. Veg..

[21] Lungu L. (1973). Analiza brioflorei din lunca Borcutului de la Cristişor-Neagra Broştenilor (Carpaţii Orientali). An. Univ. Bucureşti. Ser. Biol. Veg..

[22] Pascal P., Toma M. (1977). Contribuţii la cunoaşterea brioflorei bazinelor Suha Mare şi Suha Mică (jud. Suceava). Anu. Muz. Ştiinţe Nat. Piatra Neamţ. Ser. Bot. Zool..

[23] Ştefureac T., Pascal P. (1981). Conspectul briofitelor din Bucovina. Stud. Comunicări Ocrotirea Nat..

[24] Papp C. (1931). Contribution à la systématique des bryophyttes de la Moldavie, suivie de quelques considérations bryogéographiques. Ann. Sci. L’Université Jassy.

[25] Boros Á., Vajda L. (1967). Bryologische beiträge zur Kenntnis der Flora Transsilvaniens. Rev. Bryol. Lichénologique.

[26] Loitlesberger K. (1900). Verzeichnis der gelegentlich einer Reise im Jahre 1897 in den rumänischen Karpathen gesammelten Kryptogamen. Ann. Nat. Mus. Wien.

[27] Radian S.Ş. (1901). Contribuţiuni la flora bryologică a Romaniei. Bul. Erb. Inst. Bot. Din Bucureşti.

[28] Ştefureac T. (1951). Consideraţii bryologice asupra rezervaţiei naturale. Bul. Ştiinţific. Secţiunea Ştiinţe Biol. Agron. Geol. Geogr..

[29] Dihoru G., Ștefănuț S., Wallfisch R., Pop O.G., Pop O.G., Vergheleţ M. (2003). Bryophytes Flora of the Piatra Craiului Massif. Research in the Piatra Craiului National Park 1.

[30] Gündisch F. (1977). Beitrag zu einer Moosflora des Zibin-Gebirges. Stud. Comunicări. Ştiinţe Nat..

[31] Ştefureac T., Popescu A., Lungu L. (1959). Noi contribuţii la cunoaşterea florei şi vegetaţiei bryophytelor din Valea Lotrului. Studii şi cercetări de biologie. Ser. Biol. Veg..

[32] Péterfi M. (1904). Hunyadmegye lombosmohai. Hunyadmegyei Történelmi Régészeti Társulat Évkönyve.

[33] Györffy I. (1903). Négy ritkább növény új termőhelye Erdélyben.-Vier neue Standorte seltenerer Pflanzen in Siebenbürgen. Magy. Bot. Lapok.

[34] Péterfi M. (1908). Adatok a Biharhegység mohaflorájának ismeretéhez. Math. Természettudományi Közlemények.

[35] Goia I., Heltmann H., Von Killyen H. (2000). Moosgesellschaften des Faulen Holzen im oberen Arieş-Becken (Kreis Alba, Rumänien). Naturwissenschaftliche for-chungen über Siebenbürgen VI. Beiträge zur Geographie, Botanik, Zoologie und Paläontologie.

[36] Goia I., Schumacker R. (2003). Decaying wood communities from the upper basin of the Arieș River conserving rare and vulnerable bryophytes. Contrib. Bot..

[37] Goia I., Mătase D. (2001). Bryofloristical research in the Someşul Cald Gorges. Contrib. Bot..

[38] Goia I., Schumacker R. (2002). The Bryophytes from rotten wood in the Arieşului Mare Basin. Contrib. Bot..

[39] Ştefănuț S. (2008). Dinamica colonizării lemnului mort de către briofite în pădurile de molid. Programul de Cercetare de Excelenţă 2005–2008. Mener 2008. Mediu.

[40] Boros Á. (1941). Briológiai adatok a Kárpátokból. Bot. Közlemények.

[41] Boros Á. (1951). Der Flora von Ungarn und der Karpaten. Acta Biol. Acad. Sci. Hung..

[42] IFN (2019). The National Forest Inventory. https://roifn.ro/site/en/.

[43] Deme J., Csiky J. (2021). Development and survival of Buxbaumia viridis (Moug. ex DC.) Brid. ex Moug. & Nestl. sporophytes in Hungary. J. Bryol..

[44] Číhal L., Fialová L., Plášek V. (2020). Species distribution model for Buxbaumia viridis, identifying new areas of presumed distribution in the Czech Republic. Acta Musei Sil. Sci. Nat..

[45] Guillet A., Hugonnot V., Pépin F. (2021). The Habitat of the Neglected Independent Protonemal Stage of Buxbaumia viridis. Plants.

[46] Wiklund K. (2002). Substratum preference, spore output and temporal variation in sporophyte production of the epixylic moss Buxbaumia viridis. J. Bryol..

[47] Kropik M., Zechmeister H.G., Moser D. (2020). Climate Variables Outstrip Deadwood Amount: Desiccation as the Main Trigger for Buxbaumia viridis Occurrence. Plants.

[48] Wiklund K., Rydin H. (2004). Ecophysiological constraints on spore establishment in bryophytes. Funct. Ecol..

[49] Sabovljević M.S., Ćosić M.V., Jadranin B.Z., Pantović J.P., Giba Z.S., Vujičić M.M., Sabovljević A.D. (2022). The Conservation Physiology of Bryophytes. Plants.

[50] Infante M., Heras P. (2018). Notes on the Herbivory on Buxbaumia viridis Sporophytes in the Pyrenees. Cryptogam. Bryol..

[51] Young A.G., Port G.R., Emmett B.J., Green D.I. (1991). Development of a forecast of slug activity: Models to relate slug activity to meteorological conditions. Crop Prot..

[52] Hutchinson J.M., Reise H., Skujienė G. (2017). Life cycles and adult sizes of five co-occurring species of Arion slugs. J. Molluscan Stud..

[53] Slotsbo S., Hansen L.M., Holmstrup M. (2011). Low temperature survival in different life stages of the Iberian slug, Arion lusitanicus. Cryobiology.

[54] Spitale D., Mair P. (2017). Predicting the distribution of a rare species of moss: The case ofBuxbaumia viridis(Bryopsida, Buxbaumiaceae). Plant Biosyst. Int. J. Deal. Asp. Plant Biol..

[55] McCune B. (2007). Improved estimates of incident radiation and heat load using non-parametric regression against topographic variables. J. Veg. Sci..

[56] Majasalmi T., Rautiainen M. (2020). The impact of tree canopy structure on understory variation in a boreal forest. For. Ecol. Manag..

[57] Puletti N., Canullo R., Mattioli W., Gawryś R., Corona P., Czerepko J. (2019). A dataset of forest volume deadwood estimates for Europe. Ann. For. Sci..

[58] Öder V., Petritan A.M., Schellenberg J., Bergmeier E., Walentowski H. (2021). Patterns and drivers of deadwood quantity and variation in mid-latitude deciduous forests. For. Ecol. Manag..

[59] ESRI (2019). ArcGIS Release 10.7.1.

[60] Brown J.L., Bennett J.R., French C.M. (2017). SDMtoolbox 2.0: The next generation Python-based GIS toolkit for landscape genetic, biogeographic and species distribution model analyses. PeerJ.

[61] Fick S.E., Hijmans R.J. (2017). WorldClim 2: New 1-km spatial resolution climate surfaces for global land areas. Int. J. Climatol..

[62] Evans J.S., Oakleaf J., Cushman S.A., Theobald D. (2014). An ArcGIS Toolbox for Surface Gradient and Geomorphometric Modeling, Version 2.0-0. http://evansmurphy.wix.com/evansspatial.

[63] Naimi B., Hamm N.A., Groen T.A., Skidmore A.K., Toxopeus A.G. (2014). Where is positional uncertainty a problem for species distribution modelling?. Ecography.

[64] R Core Team (2022). A Language and Environment for Statistical Computing.

[65] Chatterjee S., Hadi A.S. (2006). Regression Analysis by Example.

[66] Naimi B., Araújo M.B. (2016). sdm: A reproducible and extensible R platform for species distribution modelling. Ecography.

[67] Eustace A., Esser L.F., Mremi R., Malonza P.K., Mwaya R.T. (2021). Protected areas network is not adequate to protect a critically endangered East Africa Chelonian: Modelling distribution of pancake tortoise, Malacochersus tornieri under current and future climates. PLoS ONE.

[68] IUCN Standards and Petitions Committee (2022). Guidelines for Using the IUCN Red List Categories and Criteria. Version 15.1. Prepared by the Standards and Petitions Committee. https://www.iucnredlist.org/resources/redlistguidelines.

[69] European Commission (2020). Communication from the Commission to the European Parliament, the Council, the European Economic and Social Committee and the Committee of the Regions. EU Biodiversity Strategy for 2030: Bringing Nature Back into Our Lives. https://eur-lex.europa.eu/resource.html?uri=cellar:a3c806a6-9ab3-11ea-9d2d-01aa75ed71a1.0001.02/DOC_1&format=PDF.

