# Supplementary material for: Population and Conservation Status of Buxbaumia viridis (DC.) Moug. & Nestl. in Romania
Source: Plants (Basel). 2023 Jan 19;12(3):473. doi: 10.3390/plants12030473 (PMC9920756; doi:10.3390/plants12030473)
Supplement: Supplementary file 1 [file plants-12-00473-s001.zip › plants-2156245-supplementary.pdf]

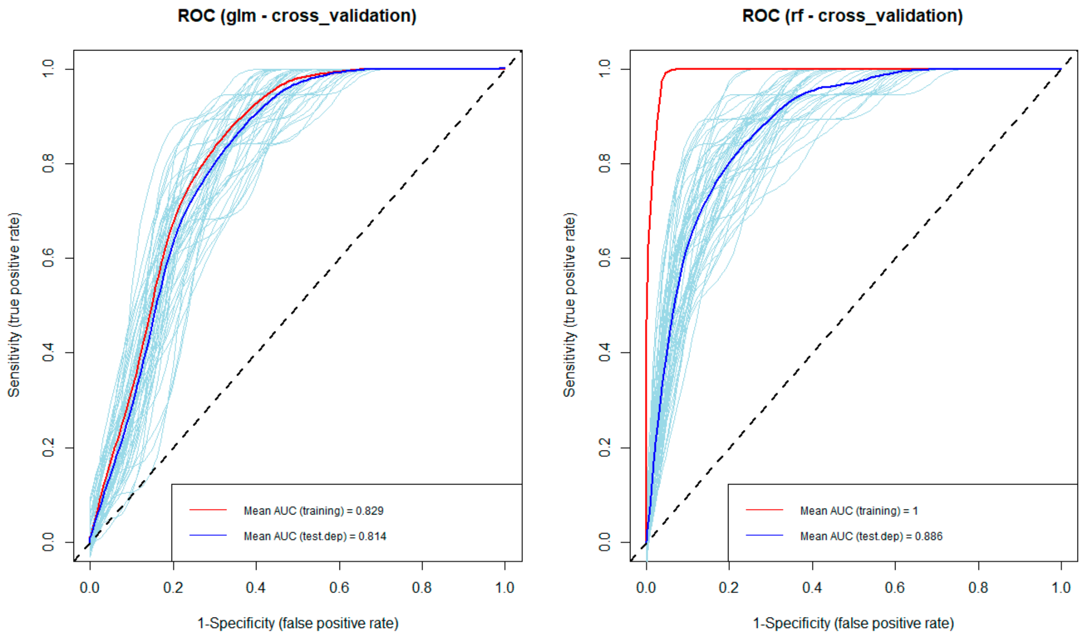

Figure S1. ROC-AUC curves for the GLM and RF models

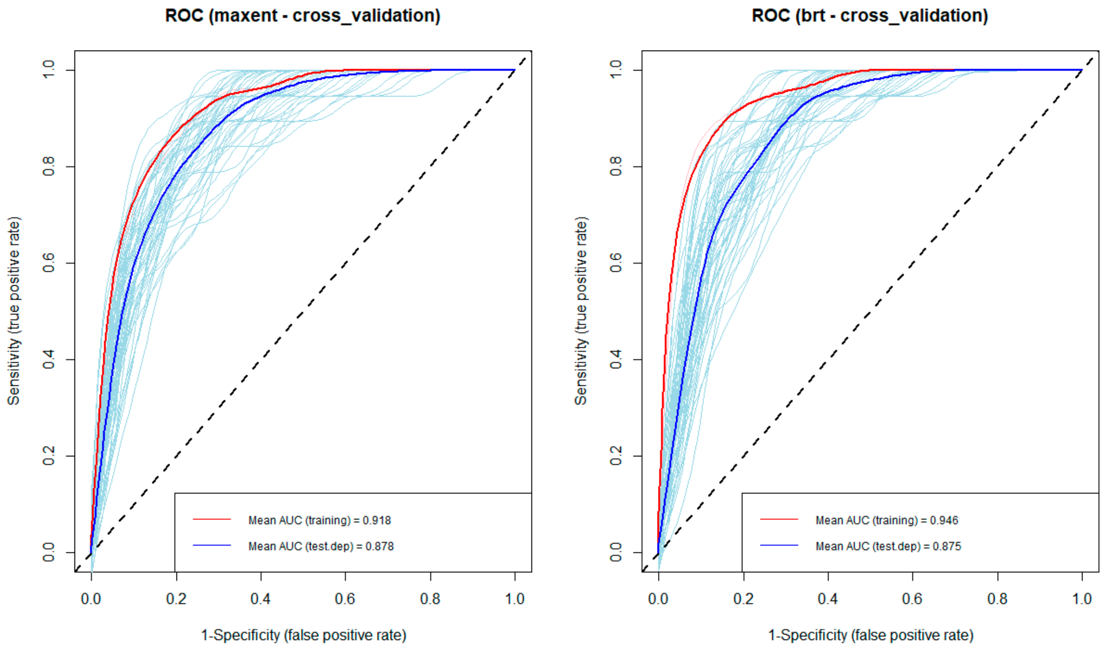

Figure S2. ROC-AUC curves for the Maxent and BRT models

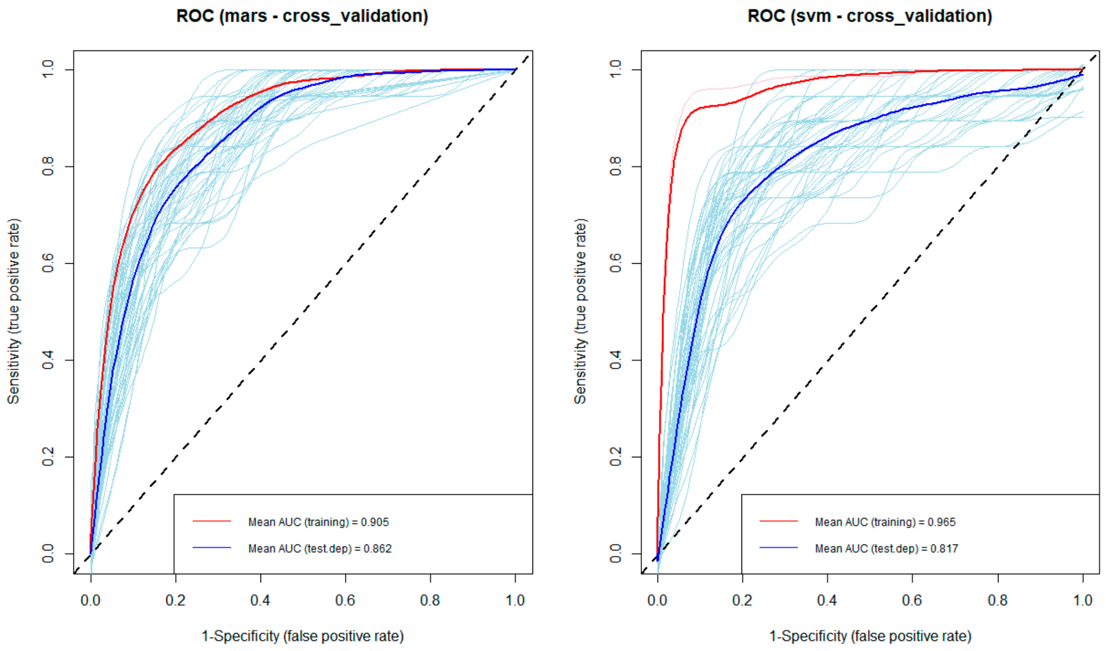

Figure S3. ROC-AUC curves for the MARS and SVM models

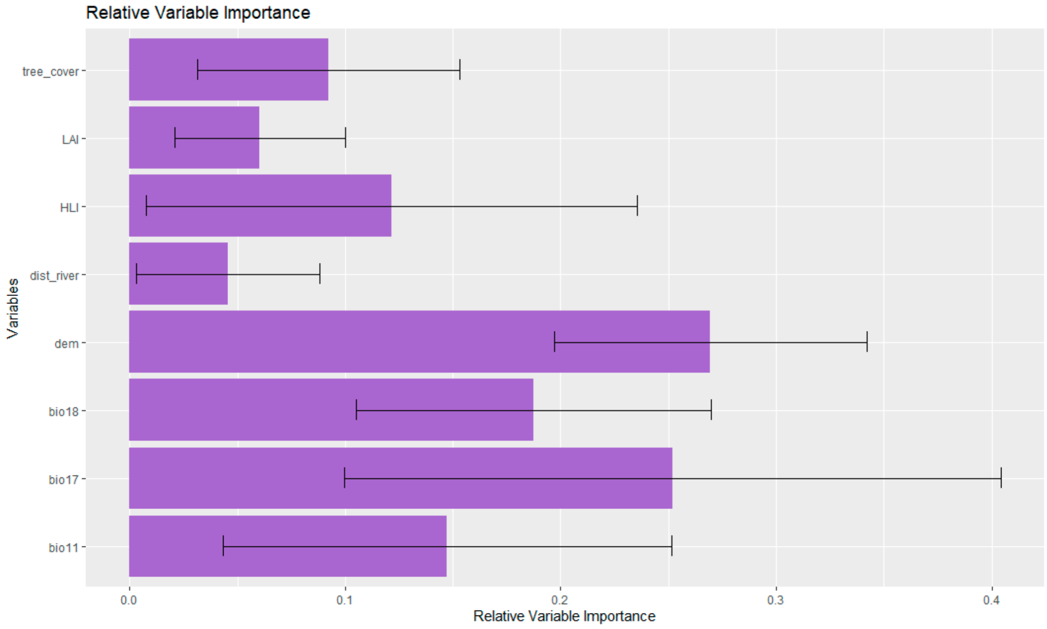

Figure S4. Relative variable importance for all models across all modeling methods (SD bars included)

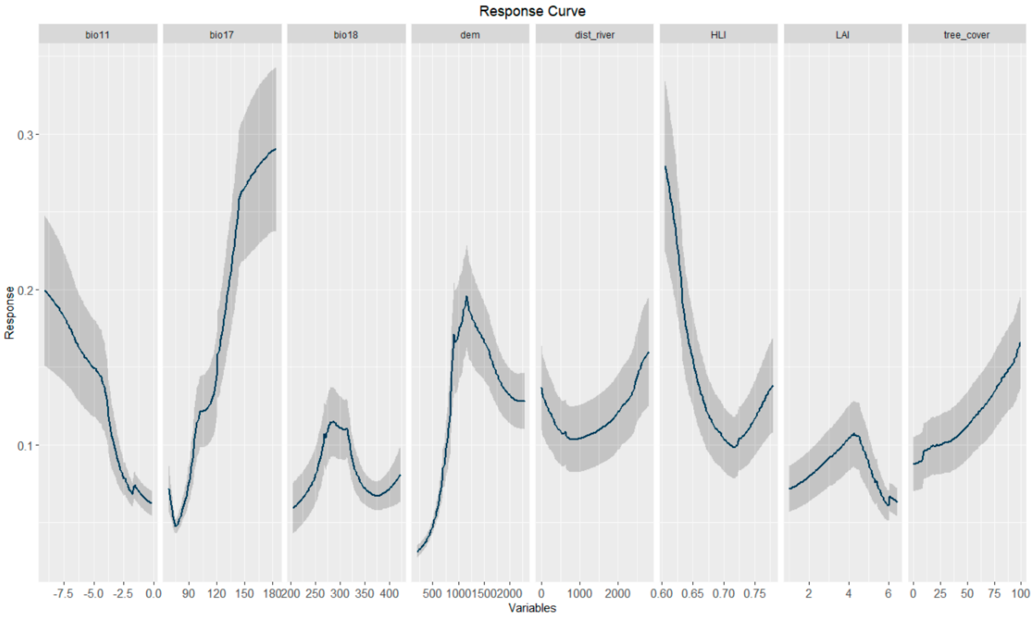

Figure S5. The response curves for all eight predictors
